# Relationship between level of empathy during residency training and perception of professionalism climate

**DOI:** 10.1186/s12909-020-02231-0

**Published:** 2020-09-21

**Authors:** Aliya B. Aziz, Syeda Kauser Ali

**Affiliations:** 1 Department of Obstetrics and Gynaecology, Aga Khan university Hospital, Stadium Road, P.O. Box 3500, Karachi, Pakistan; 2 Department of educational development, Aga Khan university Hospital, Stadium Road, P.O. Box 3500, Karachi, Pakistan

## Abstract

**Background:**

Empathy is one of the vital personality attributes for all physicians. It is essential for establishing general interpersonal relationships among doctors and patients. Unfortunately, there is evidence for the decline of physician’s empathy during the clinical training phase and is a major concern for medical educators worldwide. One of the major factors reported for the decline of this trait is an unprofessional learning environment.

**Objective:**

This study examines the relationship between empathy level and perception of climate of professionalism among residents.

**Method:**

The study participants included 70 residents of Obstetrics & Gynecology and Pediatrics departments of a private sector tertiary care hospital in Karachi, Pakistan. Two self-administered internet based surveys - Jefferson Scale of Physician Empathy (JSPE) and “Professionalism Climate Instrument”(PCI) - were administered to assess the level of empathy among the participants and their perception of professionalism in the learning environment. The relationship between the level of empathy and professionalism was analyzed using Spearman rank correlation.

**Results:**

The overall response rate was 81.4% with mean empathy level of 103 ± 13. The internal consistency of each scale measured by Cronbach’s coefficient α was 0.76 for JSPE and 0.65 for PCI. No significant difference was observed in the mean empathy scores between senior and junior residents of both specialties. Statistically significant difference in empathy scores existed between female and male residents (*p* = 0.012; 95% CI, 2.27 to 17.59). The mean PCI score was 106 + 8.88 with no significant difference among residents of two specialties. Professionalism score was not found to vary with either the year of residency or gender. Empathy score and professionalism climate were not found to be correlated (r_s_ = 0.56, *p* = 0.64).

**Conclusion:**

The findings suggested that empathy is a relatively stable trait that remains unchanged during residency training programs. Female residents had higher empathic concern than the male trainees, however, the empathy level of the participants was not found to be influenced by the climate of professionalism.

## Background

Medical professionalism lays the foundation of patient-physician trust and represents the relationship between medicine and society. Nowadays, increasing attention is being focused to develop professionalism among medical school graduates [1]. American Board of Internal medicine (ABIM) has taken a lead to address the need to promote professionalism [2]. ABIM included altruism, accountability, excellence, duty, honor and integrity, and respect for others as the elements of professionalism [1]. The Accreditation Council on Graduate Medical Education (AGGME) recommended six general competencies, such as patient care, medical knowledge, practice-based learning and improvement, professionalism, and system-based practices among the medical residents [1]. While knowledge and skill are imperative, the unique characteristic of medical professionalism is empathy [3].

### Empathy

Empathy commonly referred to as an ability to “put oneself in someone else’s shoes”, is an essential component of physician’s therapeutic effectiveness (Osler 1963). Empathy involves understanding not experiencing another’s suffering. Hojat et al. differentiate these two concepts by asserting that: “… sympathy is the act, or the capacity of entering into or joining the feelings of another person. Empathy is described as a capacity to understand but without joining the feeling of the patient” [4]. Empathy is defined as a cognitive attribute that implies an understanding of the inner experiences and perspectives of the patients’ and at the same time communicating this understanding with patients’ and supporting them [5, 6]. Studies show that empathy is linked with enhanced patient’s satisfaction, treatment compliance [7] and improved patients’ outcome [8]. The Jefferson Scale of Physician’s Empathy (JSPE) is considered as a reliable, valid, and psychometrically sound instrument to measure empathy [9, 10] [11–13].

### Professionalism climate

Over the last two decades increased attention has been given to the promotion of medical professionalism and improving institutional culture. Professionalism in clinical environment has an impact on empathy and shaping attitudes, which are manifested through the hidden curriculum [14] [15]. A simplistic way of viewing the hidden curriculum in the educational arena is to view each attending physician as a role model and each resident as a receptive learner. This learner will see the physician’s positive and negative attributes and will learn what is implicitly taught by example every day rather than the explicit teaching by lectures, tutorials and ward rounds [16].

Despite the endeavor of the institutes to improve professionalism among medical students and residents, there are major concerns about poor role modeling of professionalism as part of the hidden curriculum [14] [17].

Professionalism in the clinical environment which manifests through the hidden curriculum impacts on empathy and shapes attitudes. Assessment of climate of professionalism in learning environment is essential to gauge medical professionalism. The “climate of professionalism instrument” developed by Quaintance et al. has been successfully used in a number of studies [18]..

Reduction in empathy and compassion levels among medical students has been linked with excessive stress and burn out within the learning environment [14]. Many studies have reported high levels of stress during residency education; however, no study could be found that studied the relationship between the professionalism climate and residents’ level of empathy.

This study was conducted to measure the empathy level and to determine the relationship between the perceived climate of professionalism and the level of empathy among the residents of two specialties, which are considered highly stressful. In addition we also studied if there was any difference in the level of empathy among men and women, years of residency training and in the climate of professionalism among the two specialties.

## Method

A descriptive correlational study design utilizing survey methodology was used.

### Study participants

included residents from first to fourth year of women and child health division, comprising departments of Obstetrics & Gynecology and Pediatrics at Aga Khan University, Karachi. The total number of residents in all 4 years in the Obstetrics & Gynecology department was 23 and in the Pediatrics department was 63.

### Sample size

The sample size was calculated with “PASS 15” software having the limit of α- error less than 5% [19]. All residents in both the departments who consented and filled both JSPE and PCI survey forms were included. Residents who did not fill both the forms were excluded from the analysis. Of the total 86 residents, 70 completed both the surveys and were included in the study.

### Data collection tools

The level of empathy and professionalism climate data was collected using English version of Jefferson *Scale of Physician Empathy (JSPE)* and the *Climate of Professionalism instrument (PCI)*. JSPE is a “self-administrated 20-item instrument intended to measure empathy in the patient-care context.” It covers three underlying constructs of empathy including perspective taking (10 items), compassionate care (eight items), and standing in the patient’s shoes (two items). These items were answered on a 7-point Likert scale with 1 (strongly disagree) to 7 (strongly agree) for positive items and 1 (strongly agree) to 7 (strongly disagree) for negative items; scores range from 20 to 140. Higher scores indicate greater empathetic capacity [11].

*PCI* comprises of 12 statements on behaviors associated with professionalism in a clinical learning environment. Residents were required to rank students, residents, and attending physicians on a four-point Likert scale according to the frequency with which these behaviors were observed in each group in the past year (Mostly = 4, Often = 3, sometimes = 2, and rarely =1), for a total of 36 items. Six negatively worded items were reverse coded. The score ranged from 36 to 144. Higher scores on the PCI denote more professional behaviors in that particular group. Permission for using the instrument was obtained before use.

### Ethical considerations

The study was approved by the institutional review board of Aga Khan University Hospital (4817-DED-ERC-17). All participants were recruited on a voluntary basis and a written informed consent was obtained before participation under principles of full disclosure. Confidentiality of the participants was maintained by using anonymous surveys.

### Data collection methodology

The data was collected by web based anonymous survey in the month of October, 2017. Unique IDs were created for the respondents to match the responses on the two instruments for statistical analysis. The authorized web link provided by Thomas Jefferson University was provided to the study participants through email. The participants were provided with their unique respondent IDs required to open the survey site. The Principal investigator (PI) was blinded for these IDs to maintain confidentiality. The residents who were posted at the secondary site hospitals were contacted over phone and respondent ID was sent through email. In order to enhance the number of participants, three reminder emails were sent to the residents.

### Statistical analysis

Was done using SPSS statistical software version 19. Chi –square test and Fisher’s exact test were used for categorical variables. Level of significance was defined as *p* < 0.05 for all statistical analyses. An independent- sample t –test was performed to determine any difference in mean scores of both empathy and PCI between senior and junior residents, and male and female residents. Cronbach’s α was calculated for reliability estimates. Spearman rho analysis was done to detect the correlation between PCI and JSPE scores.

## Results

Out of total of 86 residents in women and child health division, 70 responded to both the surveys representing an 81.4%response rate. The response rate was better in Obstetrics &Gynecology department; 22 out of 23(95.65%) compared to Pediatrics; 48 out of 63(76.2%). All respondents in Obstetrics &Gynecology were females and comprised of 31.4% and remaining 68.6% belonged to Pediatrics. Most of the respondents (59%) were in the age range of 28–30 years. There was a female preponderance with 57 females (81.43%) and 13 males (18.47%). For the purpose of analysis, residents in years 1 and 2 were grouped as junior residents and in years 3 and 4 as senior residents. In both departments, majority of participants, about 70% were juniors, while only 29% were seniors (Table 1).

**Table 1:** Frequency distribution of respondents by year of residency

Residency year	Frequency	Percent	Obstetrics and Gynecology(N)	Pediatrics (N)
**Year 1**	**21**	**30.00**	**7**	**14**
**Year 2**	**28**	**40.00**	**11**	**17**
**Year 3**	**12**	**17.14**	**2**	**10**
**Year 4**	**9**	**12.86**	**2**	**7**

Cronbach’s α for JSPE scale was found to be 0.76 and for PCI was 0.65. Sub scale analysis of the items for professional and unprofessional behaviors showed Cronbach’s α of 0.8 and 0.57 respectively.

The overall mean empathy score was 103(SD 13) with a slightly higher mean for the Obstetrics & Gynecology respondents. The mean empathy score for Gynaecology residents was 106.1(SD 12.5), and for the Pediatrics respondents was 101.6 (SD 13.1).. The corresponding mean PCI scores were 100.3 (SD 8.43) and 100.5 (SD 8.88) respectively. Independent t-test indicated no statistically significant difference in mean empathy scores between junior and senior residents in both departments. (Obgyn- ***t***_**(20) =**_ **− 1.64,** ***p*** **= 0.117,** Paeds**-** ***t***_**(20) =**_**0.20,** ***p*** **= .843** and PCI scores (obgn- ***t***_**(8) =**_ **− 1.77,** ***p*** **= .115**, Paeds-) bet ***t***_**(44) =**_ **1.9,** ***p*** **= .06)**. **(**Table 2**).**

**Table 2:** Mean empathy and PCI score in OBGYN/Paediatrics based on year of residency

	Total	Obstetrics&Gynaecology		Paediatrics	
		Junior Years 1 and 2	Seniors years 3 and 4	Junior Years 1 and 2	Seniors years 3 and 4
Number of Respondents	70	18	4	31	17
**EMPATHY SCORES**					
**Range**	**73–125**	**73–119**	**106–122**	**73–120**	**80–125**
**Mean**	**103**	**104.1**	**115.0**	**101.3**	**102.1**
**PCI SCORES**					
**Range**	**85–124**	**85–114**	**101–112**	**82–124**	**86–112**
**Mean**	**100.3**	**99.2**	**105**	**101.1**	**99.0**

Overall, there were 57 female and 13male respondents in both specialties. The mean empathy score and SD of female and male participants were 104 .1(SD 12.1) and 94.9(SD 14.08) respectively. Independent t-test for female versus male residents in both specialties (***t***_(68)_ = 2.58, *p* = 0.012) revealed that the mean empathy score in female residents (9.94; 95% CI, 2.27–17.59) was much higher than the male residents.

Table 3 describes the results of resident’s perceived observations of selected professional and unprofessional behavior among medical students, residents, and faculty. There were total twelve behaviors; of them 6 professional and 6 unprofessional. The range of scores possible for both professionalism and unprofessionalism constructs was 18–24. The mean professionalism score was perceived to be highest in the resident group and lowest in the medical student group, whereas mean unprofessionalism score was similar in both faculty and the resident group and slightly higher among the students.

**Table 3:** Observed Mean Professionalism & Unprofessionalism scores by residents on PCI

	N	Mean	SD
**Professionalism**			
**Student**	**70**	**15.12**	**3.73**
**Resident**	**70**	**18.82**	**3.06**
**Faculty**	**70**	**16.7**	**3.29**
**Unprofessionalism**			
**Student**	**70**	**17.9**	**2.46**
**Resident**	**70**	**17.01**	**2.42**
**Faculty**	**70**	**17.15**	**2.48**

Spearman’s rank–order correlation indicated absence of correlation (**r**_**S** =_0.056_,_ p = 0.64) between empathy level and professionalism.

## Discussion

The current study detected that mean empathy score among medical residents in Aga Khan University was 103 ± 13 on JSPE scale. This value was relatively lower than the empathy scores observed among the residents in the Western countries, such as USA and Italy [11, 13]. However, the mean empathy score of this study was higher than Korean physicians and even comparable with Japanese and Iranian physicians, thus supporting Hojat’s hypothesis of socio cultural differences existing between Western and Asian countries [20].

The present study could not detect any significant difference in the mean empathy scores among the residents of two different specialties as opposed to the previous findings [21, 22]. One of the possible explanations for this might be that both these specialties are more ‘people- oriented’ rather than ‘technology oriented’ and thus the residents in these specialties are assumed to have direct patient interaction and might have better communication skill than their counterparts in other technology based specialties and thus have higher empathy scores [20].

The mean empathy scores among junior and senior residents of both specialties did not show significant variation. The same results were seen in a fairly recent study involving Singaporean residents where empathy levels were stable throughout the training period [23].

This study found a higher empathy level among the female residents compared to males in both specialties. This finding was in accordance with many other studies [10, 20, 24]. Probable explanation for this finding could be the association between activation of right cerebral hemisphere and empathy level in women. Another possible reason could be higher emotional receptivity in women than men, which might provide more emotional support, greater care, and also development of more interpersonal relationships. In contrast, a study by Mathew (2016) showed a higher empathy level in men and till date only one study had reported no gender based difference in empathy level [25].

The internal consistency of the PCI (Cronbach’s α of 0.65) was less than the standard value. However, dividing the items into professional and unprofessional constructs, led to an improvement of the Cronbach’s α to 0.8 for the professional construct, while for unprofessional behaviors it remained 0.57, proposing a probable shortcoming of this instrument for the sample used in this study. Moreover, use of negative words for ‘unprofessionalism’ items might have caused some problems in interpretation. Similar problem was confronted by Spiwak, who performed exploratory analysis to identify these professional versus unprofessional behaviors [26]. This would occur due to differences in population and probably there is a need for a different instrument to measure this important construct.

The most obvious finding of this study was the climate of professionalism existing in the institution. No significant difference was detected by the residents in the mean observed unprofessionalism behaviors among three groups i.e., medical students, residents and faculty, but a higher professionalism was detected among the residents. The finding was in accordance to the study by Quaintance on American medical learners showing a significant difference between preclinical and clinical trainee’s observations of professionalism among students, residents, and faculties [18]. Spiwak reported that residents rated the faculty to be the poorest in terms of observed professional behaviors [26]. The finding of this study can be explained as the medical residents might rate the peer group more favorably since they had social similarity in behaviors and characteristics [26]. Moreover, the residents spent more time in training, had increased opportunity to interact with the faculties, who act as a role model for the trainees and they learnt professionalism and unprofessionalism behaviors by observing the faculty [27, 28] [17].

The current study could not identify any correlation between empathy score and professionalism climate. This was in opposition to the study by Brazeau who found a direct correlation between empathy score and PCI score among medical students in the learning environment [14]. The probable reason for this finding could be the problem of self-reporting of empathy level by the residents and also low reliability of PCI instrument. Also Brazeau’s study included medical students only and the current study included residents directly providing patient care, with long working hours and sleep deprivation, probable causes of lower level of empathy in the clinical training [14].

### Limitations

There were some limitations of this study. Firstly, the use of self-reported questionnaires for detection of empathy level might not reveal the actual empathetic behavior of the residents during medical practice; rather it would indicate residents’ orientation towards empathy. Secondly, the external validity or generalization of the findings was limited due to convenient sampling that included residents of a single institution of a particular geographical area. Thirdly, the sample size was small with only residents of two specialties leading to inadequate statistical power. Fourthly, since it was not a longitudinal study, it was difficult to ascertain empathy decline among the residents. Last, but not the least, PCI instrument despite being a validated tool for measuring professionalism climate, might be unable to capture participants’ evaluation of others’ behavior properly in this study.

## Conclusion

In summary this is the first study to examine the empathy level and professionalism climate and correlation between the two among the medical residents in a developing country. Despite the aforementioned limitations, this work offers invaluable understanding about empathy and professionalism in the learning environment of the residents. The study suggested that empathy is a relatively constant quality among the sample of residents under study that remained unaffected by year of residency or specialty. However, the empathy level of the female residents was found to be higher than male residents. The study could not detect a significant correlation between professionalism climate and empathy level.

Future research should be directed towards developing a longitudinal study involving a large number of residents from different specialties and different medical institutions to gain more insight into the relationship between professionalism climate and empathy and for a possible change in empathy level with progression in training years.

## Abbreviations

ACGME: American Council of Graduate Medical Education
PCI: Professionalism climate instrument
ABIM: American board of internal medicine
JSPE: Jefferson Scale of Physician Empathy
